# HCMV Envelope Glycoprotein Diversity Demystified

**DOI:** 10.3389/fmicb.2019.01005

**Published:** 2019-05-15

**Authors:** Mathilde Foglierini, Jessica Marcandalli, Laurent Perez

**Affiliations:** ^1^Faculty of Biomedical Sciences, Institute for Research in Biomedicine, Università della Svizzera Italiana, Bellinzona, Switzerland; ^2^Swiss Institute of Bioinformatics, Lausanne, Switzerland

**Keywords:** human cytomegalovirus, envelope glycoproteins, viral diversity, multiple sequence alignment, protein sequence analysis, phylogenic analysis

## Abstract

Human cytomegalovirus (HCMV) is the leading viral cause of congenital birth defects and is responsible for morbidity and mortality in immunosuppressed individuals. Considerable efforts have been deployed over the last decade to develop a vaccine capable of preventing HCMV infection. However, in recent clinical trials, vaccines showed at best modest efficacy in preventing infection. These findings might be explained by the high level of sequence polymorphism at the genomic level. To investigate if genomic variation also leads to antigenic variation, we performed a bioinformatic sequence analysis and evaluated the percentage of conservation at the amino acid level of all the proteins present in the virion envelope. Using more than two hundred sequences per envelope glycoprotein and analyzing their degree of conservation, we observe that antigenic variation is in large part limited to three proteins. In addition, we demonstrate that the two leading vaccine candidates, the pentamer and gB complexes, are well conserved at the amino acid level. These results suggest that despite genomic polymorphism, antigenic variability is not involved in the modest efficacy observed in the recent clinical trials for a HCMV vaccine. We therefore propose that next-generation vaccines should focus on stabilizing and refining the gB domains needed to induce a protective humoral response.

## Introduction

Human cytomegalovirus (HCMV) is a member of the *Betaherpesvirinae* subfamily of *Herpesviridae* and 40–80% of the human adult population is seropositive to HCMV (Kenneson and Cannon, 2007). Primary infection is generally asymptomatic in immunocompetent individuals. Nonetheless, HCMV establishes lifelong latency in infected individuals (Zhuravskaya et al., 1997), and viral reactivation in immunosuppressed hosts such as transplant patients and patients with AIDS can cause severe diseases or lead even to death (Rando et al., 1990). HCMV is also the major cause of congenital birth defects (0.7% of the newborns) (Kirby, 2016), exceeding fetal alcohol syndrome and Zika virus infection in industrialized countries. Current antiviral therapies and treatments with hyperimmune globulin transfusion to control viremia are not efficient (Tandon and Mocarski, 2012). Due to the prevalence, severity and importance of this virus, the United States National Academy of Medicine considers that obtaining an effective vaccine against HCMV is a top priority in public health. However, HCMV is a complex virus with a genome encoding at minimum 170 open reading frames (ORFs) (Stern-Ginossar et al., 2012) and expressing at least nineteen membrane proteins integral to the virion envelope (Weekes et al., 2014). These envelope proteins are primarily required for binding to the host cells, viral entry and, in some cases, immune evasion by sequestering human chemokines (Frascaroli et al., 2006; Frascaroli et al., 2018). HCMV broad cellular tropism (epithelial/endothelial cells, fibroblasts, monocytes/macrophages, smooth muscle cells, neurons, stromal cells and hepatocytes) is due to two glycoprotein complexes responsible for the interaction with specific host cell receptors (Malito et al., 2018; Nguyen and Kamil, 2018). The gHgLgO (trimer) complex is required for infection of all cell types and is known to bind the platelet-derived growth factor receptor α (PDGFRα) expressed on fibroblasts (Kabanova et al., 2016). The gHgLpUL128pUL130pUL131A (pentamer) complex is required for viral entry in epithelial, endothelial and myeloid cells and binds Neuropilin2 (Nrp2) (Martinez-Martin et al., 2018). Membrane fusion between the virus and the surface of the host cell is catalyzed by the glycoprotein B (gB), a class III fusion protein forming a homotrimer (Burke and Heldwein, 2015; Chandramouli et al., 2015). Promising HCMV vaccine candidates have been sought among the viral proteins present in the envelope and in the tegument layer of the virus (Anderholm et al., 2016). Current vaccine research is focusing on two protein complexes of the virion envelope: the gB homotrimer and the pentamer (Chiuppesi et al., 2017; Wussow et al., 2018). Both complexes were identified as dominant targets of the humoral immune response upon infection (Macagno et al., 2010; Kabanova et al., 2014). Research studies on gB are more advanced compared to the pentamer, and a vaccine was already tested in phase I and II clinical trials (Baraniak et al., 2018a; Nelson et al., 2018b; Schleiss, 2018). The vaccine demonstrated around 50% efficacy in preventing HCMV acquisition in seronegative women (Baraniak et al., 2018a; Nelson et al., 2018a). However, antibodies elicited by the vaccine were only poorly neutralizing (Baraniak et al., 2018a; Nelson et al., 2018b). To explain these results, several hypotheses have been proposed: the presence of an undesired antigenic domain (AD) in the gB antigen used in the vaccine, the post-fusion conformation of the antigen injected, but also the sequence diversity observed among the gB proteins expressed by the different natural circulating HCMV strains (Renzette et al., 2011, 2017). Indeed, HCMV is described as being a highly polymorphic virus at the genomic level (Renzette et al., 2016), and it was shown that sequence diversity of some proteins impacts the infection outcome (Renzette et al., 2011; Galitska et al., 2018). To investigate if HCMV genomic diversity is also leading to antigenic variation of the proteins belonging to the virion envelope, we used datasets from publicly available HCMV genomes and performed sequence diversity analysis.

## Materials and Methods

### Sequences Analysis of HCMV Proteins

Eighteen envelope and membrane protein sequences of HCMV were obtained from the National Center for Biotechnology Information (NCBI) Entrez Protein database as of September 18, 2018 (Sayers et al., 2019) and were derived from the following genes: RL10, UL1, UL4, UL33, UL55, UL73, UL74, UL75, UL78, UL100, UL115, UL116, UL128, UL130, UL131A, UL132, US27, and US28. For each protein, the sequences were purged of duplicates for a given strain, partial sequences (+/- 5 amino acids) and sequences lacking the name of the strain, the country where it was isolated and/or the collection date. For downstream analysis, we used on average 240 sequences for each HCVM protein. Protein sequences were aligned with Clustal Ω (Sievers et al., 2011). For a given protein, a matrix presenting the percentage of amino acids identity between strains was generated followed by hierarchical clustering using the Heatmap function from the “ComplexHeatmap” R package (Gu et al., 2016) (Euclidean metric and complete aggregation method). The overall mean distances (percentage of mean identity) were computed with MEGA X (Kumar et al., 2018) with default parameters. Analyses of the glycoprotein pentamer complex gHgLpUL128pUL130pUL131A (UL75-UL115-UL128-UL130-UL131A) and gB (UL55) were conducted by concatenation of each glycoprotein for a given strain. We used the sequences of 207 strains containing the pentamer and gB to perform an evolutionary analysis. The evolutionary history was inferred by using the Maximum Likelihood method based on the JTT matrix-based model (Jones et al., 1992), bootstrapped 500 times. The trees with the highest log likelihood are shown and are drawn to scale, with branch lengths measured in the number of substitutions per site. Evolutionary analyses were conducted in MEGA X. Trees were visualized with iTOL (Letunic and Bork, 2016). Histograms of consensus sequence for the “hot-spots” of pentamer, gB, gN, and gO were obtained by Jalview software (Waterhouse et al., 2009). The list of HCMV strains used in this study is available as (Supplementary Table S1).

## Results

### Envelope Protein Required for Viral Tethering Are Well Conserved

**FIGURE 1:**
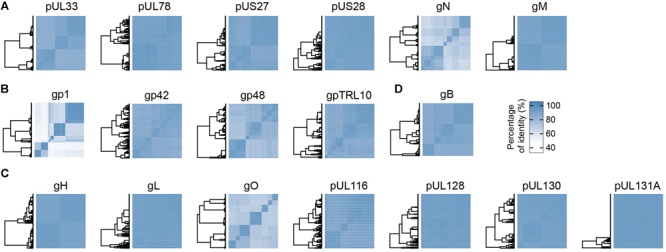
Conservation matrices of HCMV proteins integral to the virion envelope. Percentage of amino acids identity is represented as matrices using hierarchical clusters from the “ComplexHeatmap” R package. The following proteins are represented: **(A)** Four viral G protein-coupled receptors (UL33, UL78, US27, and US28) and two glycoproteins (gN and gM); **(B)** Glycoproteins with unknown function gp1, gp42, gp48, and gpTRL10; **(C)** Protein UL116, proteins composing the pentamer (gH, gL, pUL128, pUL130, and pUL131A) and the trimer (gH, gL, and gO) complexes; **(D)** gB.

Human cytomegalovirus envelope contains four viral G protein-coupled receptors (vGPCRs), namely pUL33, pUL78, pUS27, and pUS28 proteins (de Munnik et al., 2015). Among these four vGPCRs, only pUS28 is functional and possesses structural similarity to the human chemokine receptors CCR1 and CCR5 (Gao and Murphy, 1994). The pUS28 also binds human CX3CL1 and additional inflammatory chemokines from the CC-family (Krishna et al., 2018). Analysis by similarity matrix demonstrated that all vGPCRs are very well conserved (Figure 1A), a result consistent with the conserved architectural structure of the GPCRs (Congreve et al., 2017). The vGPCRs have a limited immunogenicity, due to the minimal exposure of the short loops that link the seven-(pass)-transmembrane domains. The glycoprotein M (UL100) together with glycoprotein N (UL73) forms the gM/gN heterodimer, which is one of the most abundant protein complexes within the HCMV envelope. Furthermore, this heterodimer is one of the few envelope proteins conserved among herpesviruses, thus suggesting an important function in their biology. Although the latter is not fully understood, the complex was shown to be involved in the initial interaction phases with host cells by binding heparin present on the cellular envelope (Compton et al., 1993). Interestingly, whereas gM is extremely conserved with 99% mean identity across strains, gN is one of the less conserved proteins with only 81% of identity (Figure 1A). Moreover, gN is extensively modified by O-linked sugars which contribute to its mass for more than 40 kDa (Pignatelli et al., 2001). Nevertheless, gN was identified as a target of neutralizing antibodies (Shimamura et al., 2006), a surprising result considering its sequence variability and glycosylation status (Supplementary Figure S1a).

### Structural Components of the Virion Envelope Exhibit a Low Degree of Conservation

The organization of the viral proteins in the envelope is still poorly characterized and understood. Most of the proteins from this group display a very low degree of conservation mean identity across strains (Figure 1B). The gp1 (UL1) is a glycoprotein with 224 amino acids and whose function remains unclear. However, it was shown that gp1 contains an Ig-like domain, and disruption of gp1 in HCMV generates viral growth defects in epithelial cells but not in fibroblasts (Shikhagaie et al., 2012). The gp48 (UL4) is a 48 kDa glycoprotein whose expression is non-essential for HCMV replication in cell cultures (Alderete et al., 2001). The gp42 (UL132) is a highly glycosylated protein of 270 amino acids, described to be required for cellular endocytosis of the virus. Furthermore, gp42 is necessary for optimal replication of the virus (Kropff et al., 2010). The gpTRL10 (RL10) is a 170-amino acid-long glycoprotein with no function yet identified.

### Sequence Diversity of HCMV Glycoproteins Involved in Viral Entry

Human cytomegalovirus uses two viral ligands for specific and high affinity interactions with the host cells. The pentamer complex is required for viral entry in epithelial, endothelial and myeloid cells (Ryckman et al., 2008a,b), and it represents the main target of neutralization against HCMV, eliciting extremely potent neutralizing antibodies (Macagno et al., 2010; Kabanova et al., 2014). Sequence analysis of the pentamer subunits reveals an extremely high level of conservation, with a mean identity of 98% (Figure 1C). This degree of conservation could be explained by the complex folding required to assemble the five subunits together (Chandramouli et al., 2017). Moreover, the multiple amino acid contacts occurring between the Nrp2 receptor and the gL, pUL128, pUL130, and pUL131A subunits also limit the possibility of antigenic drift (Martinez-Martin et al., 2018). Surprisingly, the trimeric complex composed of gH, gL, and gO possesses a low mean percentage of identity. The gO subunit is responsible for this low degree of conservation, since it is one of the less conserved envelope glycoproteins of HCMV, with a mean identity of 81% (Figure 1C). Interestingly, most of the sequence divergence is located in the N-terminus part of the protein (Supplementary Figure S1b), which is supposed to interact with PDGFRα, the cellular receptor of this complex (Stegmann et al., 2017, 2019). Of note, the pUL116 protein was recently identified as a binding partner for gH, forming a dimeric complex of unknown function (Calo et al., 2016). The relatively high degree of conservation observed for pUL116 might indicate that the gH/pUL116 complex interacts with host or other viral proteins. The gB protein is encoded by the highly polymorphic gene UL55 (Paterson et al., 2002). However, our sequence analysis revealed a mean identity of 96% at the amino acid level, indicating that the protein is well conserved across strains (Figure 1D). The glycoprotein B is necessary for entry in all cell types, making it an appealing candidate for a HCMV vaccine.

### Sequence Diversity of Vaccine Candidates

**FIGURE 2:**
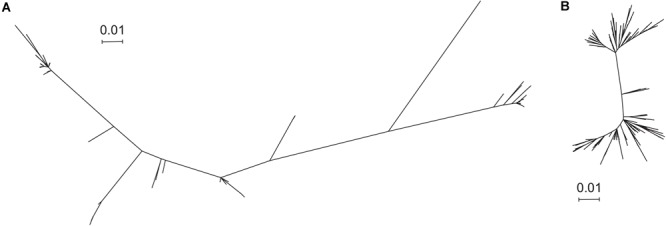
Phylogenic tree of gB and the pentamer complexes. 207 HCMV strains containing both gB and the proteins composing the pentamer were used for analysis. **(A,B)** Phylogenic analysis of the amino acid sequences by Maximum Likelihood method, bootstrapped 500 times, conducted by MEGA X. **(A)** gB tree and **(B)** pentamer complex tree.

The trimer gHgLgO is necessary for cellular entry in all cell types and represents a potential target for vaccine design. Nonetheless, the complex glycan shield and the important sequence variation of gO are likely to represent a major problem for vaccine development. The gHgL heterodimer represents an interesting option for vaccine design, since both subunits are well conserved and characterized. However, immunization experiments in animal models generated suboptimal neutralization titers (Kabanova et al., 2014). Therefore, the gB homotrimer and the pentamer represent the best option for a prophylactic HCMV vaccine either alone or in combination. Some reports suggested that gB or pentamer sequence variation could be an issue for the generation of an efficient vaccine (Renzette et al., 2011). To gain further insights into gB and pentamer sequence variability, we used a total of 207 sequences per protein, obtained from the NCBI Entrez Protein database corresponding to different strains. This allowed us to generate a phylogenic tree based on the Maximum Likelihood method for both gB (Figure 2A and Supplementary Figure S2) and the pentamer (Figure 2B and Supplementary Figure S3). This analysis demonstrated that gB sequences are more diverse among strains (Figure 2A and Supplementary Data S1) compared to the pentamer (Figure 2B and Supplementary Data S2).

**FIGURE 3:**
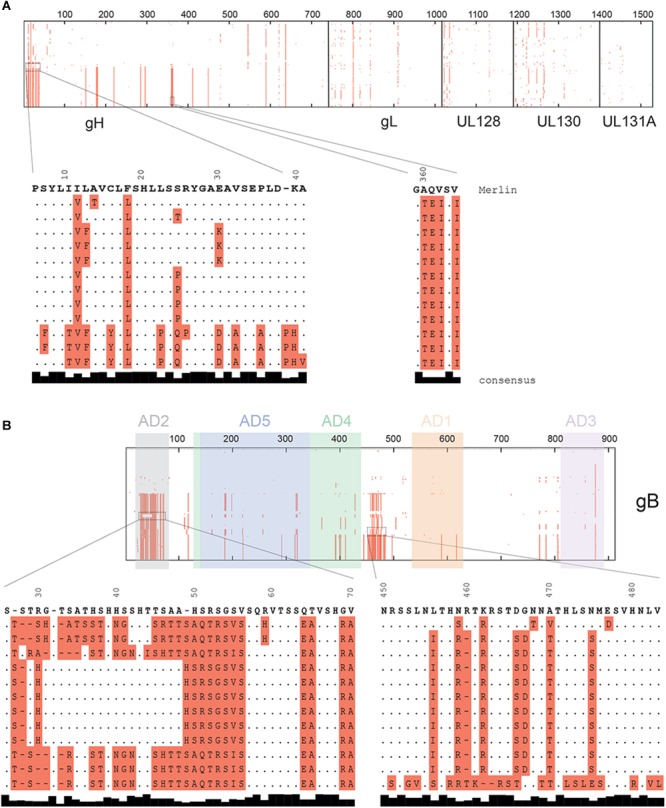
Sequence alignment of the pentamer and gB complexes. Protein sequences were aligned with Clustal Ω. All amino acids different from the reference HCMV strain (Merlin) are highlighted in red. Thick lines inside the aligned sequences represent 100 amino acids. Regions with high sequence diversity are expanded and shown in detail below the pictographic alignment. **(A)** Protein alignment of concatenated subunits of the pentamer complex (gHgLpUL128pUL130pUL131A). **(B)** gB protein alignment where AD-2 and Furin cleavage site are expanded. Antigenic domains (AD) are highlighted in different colors.

Indeed, analysis of sequence alignment demonstrated that the pentamer is well conserved, with only one main sequence variation localized on a short stretch of 30 amino acids (7 to 37) (Figure 3A) corresponding to the gH signal peptide (Chandramouli et al., 2017). In contrast, sequence variations on gB were localized on two “hot spots” (Figure 3B). These regions correspond to amino acids 27 to 70 and amino acids 451 to 483. The distal region (aa 451–483) includes the Furin cleavage site (Singh and Compton, 2000), and sequence variation of this site was initially used to classify different gB genotypes (Chou and Dennison, 1991). However, the relevance of the latter is not entirely clear concerning the infectivity and classification of HCMV strains (Stangherlin et al., 2017). The proximal region (aa 27–70) includes AD-2, which is one of the five antigenic domains (AD-1, -2, -3, -4 and -5) identified for gB. Interestingly, only antibodies directed against AD-2, AD-4, or AD-5 are able to neutralize the virus. Recently, a publication demonstrated that anti-AD-2 serum titers correlate with protection from viremia (Baraniak et al., 2018b), information that is highly relevant for vaccine design. Surprisingly, the crystal structure obtained from gB in its post-fusion conformation (Burke and Heldwein, 2015; Chandramouli et al., 2015) was not performed on the full extracellular domain and did not include the AD-2 sequence. The latter was removed, as it was believed to be unstructured and flexible in the post-fusion conformation adopted by gB.

## Discussion

Human cytomegalovirus is the most common cause of viral-induced birth defects in industrialized countries. There is no treatment or vaccine clinically available today. The virion envelope protein gB is essential for viral entry and represents a major target of the humoral immune response following infection. gB vaccines were found to be safe and immunogenic in clinical phase I and II studies, eliciting an IgG response with a gB-binding magnitude comparable to that of a natural infection (Baraniak et al., 2018a; Nelson et al., 2018b). However, despite the gB titers induced, only a minimal virus-neutralizing response was observed (Schleiss, 2018). The immunological reasons for these results are not fully understood yet, but several hypotheses have been proposed. The antigen employed comprised the intraluminal region of the gB molecule, a domain that generated non-neutralizing antibodies since it is not exposed onto the surface of the virion (Schleiss, 2018). The sequence of gB used in the vaccine is derived from a laboratory viral strain that possesses neither the tropism nor the virulence of clinical isolates (Nelson et al., 2018a). In addition, the sequence diversity observed at the genomic level was proposed as a potential issue for vaccine development (Renzette et al., 2017). Here, we performed sequence analyses for all the envelope proteins of HCMV and found that most of the proteins with a relevant function are well conserved, with the exception of gN and gO. While we investigated the relevance of antigenic variation for vaccine design, we found that the pentamer is highly conserved across all HCMV strains with a deposited sequence. The gB antigen presents two main regions of variability across strains: the Furin cleavage site and part of AD-2. However, the Furin cleavage site is not supposed to be antigenic (Potzsch et al., 2011), and only the conserved site of AD-2 is responsible for the generation of neutralizing antibodies (Baraniak et al., 2018b,c). Our analysis shows that both the pentamer and gB are well conserved across strains. We hypothesize that the modest efficacy observed in the recent clinical trials for an HCMV vaccine (Baraniak et al., 2018a; Nelson et al., 2018b) does not result from HCMV genomic polymorphism. The absence of a strong neutralizing activity induced by the gB vaccine can be explained by the immunodominant response generated against AD-1 and AD-3. It has been shown that anti-AD1 antibodies do not correlate with protection (Baraniak et al., 2018b), and the humoral response against AD-1 upon natural infection generates a very modest neutralizing activity (Speckner et al., 1999). Moreover, AD-1 is also thought to mask AD-2, and part of the latter is the target of neutralizing antibodies (Baraniak et al., 2018b). Regarding AD-3, this domain is present in the lumen of the virus; therefore, as previously discussed, it is unlikely that antibodies generated against this domain will demonstrate a neutralizing activity (Schleiss, 2018). In summary, we speculate that engineering a gB vaccine lacking the antigenic domains AD-1 and AD-3 might generate a higher neutralizing titer in comparison to the vaccine candidate used in clinical trials.

## Data Availability

All datasets analyzed for this study can be obtained by contacting the corresponding author.

## Author Contributions

MF designed and performed the bioinformatic analysis and wrote the manuscript. JM analyzed the data and wrote the manuscript. LP conceived the study, analyzed the data and wrote the manuscript.

## Conflict of Interest Statement

The authors declare that the research was conducted in the absence of any commercial or financial relationships that could be construed as a potential conflict of interest.

## Supplementary Material

FIGURE S1 Sequence alignment of gN and gO proteins. Amino acids different from the reference HCMV strain (Merlin) are highlighted in red. Thick lines inside the aligned sequences represent 100 amino acids. (**a**) Protein alignment of gN. (**b**) Protein alignment of gO.

FIGURE S2 Labeled gB phylogenic tree. Phylogenic analysis of the amino acid sequences by Maximum Likelihood method for gB with name of the strains. Bootstrap values equal or higher than 70% are shown.

FIGURE S3 Labeled pentamer phylogenic tree. Phylogenic analysis of the amino acid sequences by Maximum Likelihood method for the pentamer with name of the strains. Bootstrap values equal or higher than 70% are shown.

TABLE S1 HCMV strains used for the study.

DATA S1 gB alignment file in fasta format.

DATA S2 Pentamer alignment file in fasta format.
