# Supplementary figures and images for: HCMV Envelope Glycoprotein Diversity Demystified
Source: Front Microbiol. 2019 May 15;10:1005. doi: 10.3389/fmicb.2019.01005 (PMC6529531; doi:10.3389/fmicb.2019.01005)

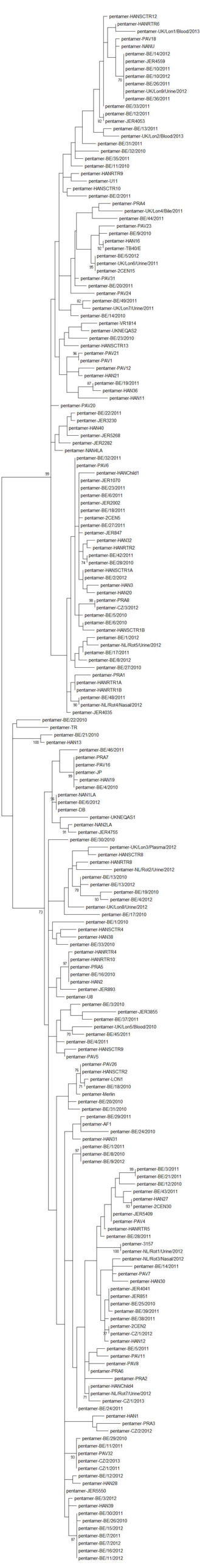

Supplement: FIGURE S1 — Sequence alignment of gN and gO proteins. Amino acids different from the reference HCMV strain (Merlin) are highlighted in red. Thick lines inside the aligned sequences represent 100 amino acids. (a) Protein alignment of gN. (b) Protein alignment of gO. [file Image_2.pdf]

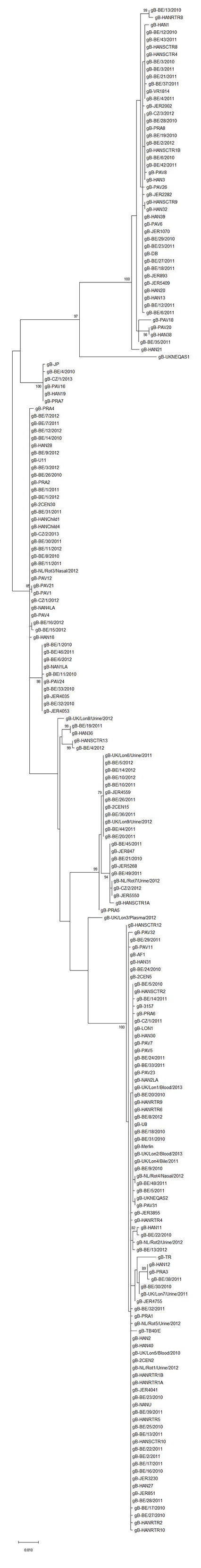

Supplement: FIGURE S2 — Labeled gB phylogenic tree. Phylogenic analysis of the amino acid sequences by Maximum Likelihood method for gB with name of the strains. Bootstrap values equal or higher than 70% are shown. [file Image_2.tif]

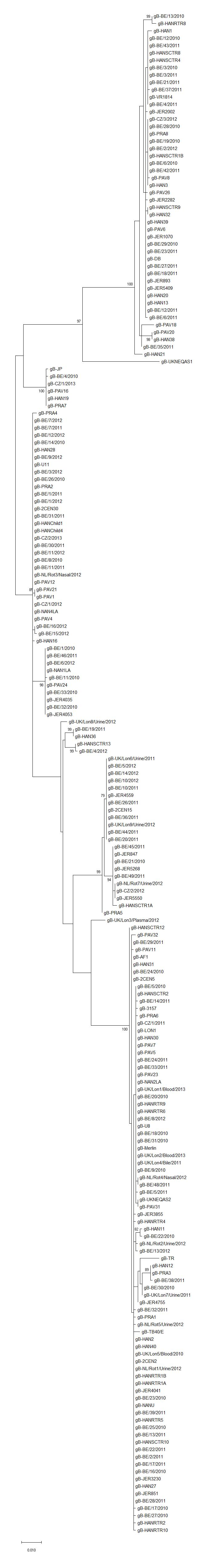

Supplement: FIGURE S3 — Labeled pentamer phylogenic tree. Phylogenic analysis of the amino acid sequences by Maximum Likelihood method for the pentamer with name of the strains. Bootstrap values equal or higher than 70% are shown. [file Image_3.TIF]
