# Supplementary material for: HCMV Envelope Glycoprotein Diversity Demystified
Source: Front Microbiol. 2019 May 15;10:1005. doi: 10.3389/fmicb.2019.01005 (PMC6529531; doi:10.3389/fmicb.2019.01005)
Supplement: TABLE S1 — HCMV strains used for the study. [file Presentation_1.pdf]

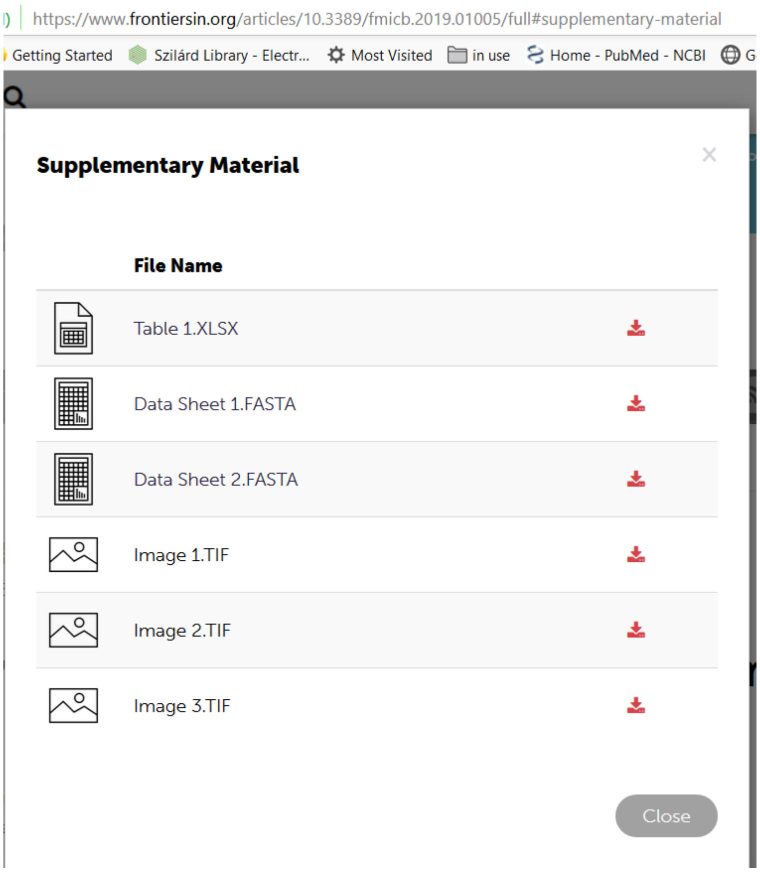

Table 1 should be labeled as Table S1

Data Sheet 1 should be labeled Data S1

Data Sheet 2 should be labeled Data S2

Image 1 should be labeled as supplementary Figure 1

Image 2 should be labeled as supplementary Data 1 and updated

Image 3 should be labeled as supplementary Data 2 and updated
